# Determination of the Strength of Consolidated Powder Materials with a Pull-Based Tester

**DOI:** 10.3390/ma16093557

**Published:** 2023-05-06

**Authors:** Justyna Wajs, Joanna Wiącek, Józef Horabik, Mateusz Stasiak

**Affiliations:** Institute of Agrophysics, Polish Academy of Sciences, Doświadczalna 4, 20-290 Lublin, Poland

**Keywords:** powder tester, consolidated powders, powder materials, mechanical properties, powder quality

## Abstract

In recent years, there has been increasing interest in the agglomeration of bulk materials. New methods are being sought to improve the measurement of bulkiness in food powders. This study aimed to design a new measuring device to assess the phenomenon of caking as well as the degree of strength of free-flowing powders. Wheat flour and potato starch have been used in the experiment and loaded into a perforated container. A steel or polypropylene measuring rod has been placed in the middle, and 5 kPa and 10 kPa were loaded, respectively. The new method is based on measuring the force through a sensor when pulling out measuring rods from the powder sample. It was shown that higher strength values have been obtained for powders loaded with 10 kPa and that groove depth has not been significant for wheat flour. Additionally, a significant difference in the evolution of the pulling force with time has been observed for wheat flour and starch, revealing a slip-stick phenomenon in the latter one. The pull-based tester is characterized by fast measurement and easy analysis of the results. The tests performed for potato starch and wheat flour have provided significantly different temporal evolutions of the pulling force.

## 1. Introduction

Powder materials constitute a wide group as raw materials and also as final products in many industries. At each stage of the technological process involving powders, special attention should be paid to production conditions. Any change in the process conditions may result in changes in the behavior of the powders, providing undesirable effects during pouring, transport, or storage of these materials [1].

Among undesirable phenomena resulting from incorrectly selected process parameters are segregation, attrition, or caking [2]. In recent years, the phenomenon of caking has attracted the attention of scientists. The particles of free-flowing powder begin to combine with each other, forming a kind of bridge, resulting in the formation of agglomerates. The deformation of the particles increases the contact area between neighboring particles, reduces the distance between them, and, consequently, may enhance caking [3]. Depending on the environmental conditions and the internal properties of the powder, agglomerates may be either easily broken or their destruction may require the application of a much higher force [4]. The determination of agglomerate strength is of high importance in many branches of industry.

There are a lot of mechanical tests and measuring methods that can be used to examine the properties of powders. The most commonly applied method, as proposed by Jenike [5], is a shear test. It is used for the determination of the strength and powder flowability properties of powders, which are of high interest to silo and process designers [6]. Based on the shear test, new instruments are being developed to examine the properties of granular materials at various levels of consolidation [7,8,9]. However, the assessment of powder flowability with the Jenike tester is time-consuming and complicated, and the analysis of the results provided by the apparatus is difficult and requires highly qualified staff [10]. Therefore, there is an urgent need to search for new and less complicated devices for assessing the flowability and caking properties of powders.

The second is a penetration test based on measuring the stress required for a penetrometer to pass through a lumpy sample. This method is very useful as an indication of the onset of caking on the surface of the powder layer [11,12]. It is widely used to assess caking in common food powders, such as wheat flour, potato starch [13,14], powdered milk, or powder mixes [15].

Modifications provided to the punch test by Hassanpour and Ghadiri [16] allowed them to develop a ball indentation technique that was applied to measure the hardness of the compressed powder. To determine the strength and caking properties of powders, Bode et al. [17] used a rheometer that measured the vertical stress required to rotate the rotor as it moved down through the caked sample. Billings, Bronlund, and Paterson [12] have used a blow tester, where a powerful stream of air is used. In this method, the speed of the compressed air gradually increases until the particles are removed from the powder bed. The air velocity at which the particles move through the air correlates with the strength of a caked sample.

The degree of caking may also be measured in a tensile test. The tensile strength of a lumpy sample is defined as the stress required to break the two halves of the split cylinder containing the caked material [18]. A similar technique has been used by Stasiak et al. [10], where the breaking strength of a column of consolidated starches was determined in a divided cylinder. Salehi et al. [19] have developed a new Greenwich Caking Tester for powders characterized by low lumps and low hardness. It is characterized by a low ratio of the chamber height to its diameter, and the measurement method is based on the displacement of the probe at a constant speed. The latest methods also focus on the development of targeted removal positions. One of them is a homemade piece of equipment called OLAF (Open-Loop Air Flow). It is equipped with special air flow chambers where the powder sample is placed. It was found that the caking occurs faster in the conditions prevailing in the test device [20]. Among the methods frequently used to determine the tensile strength of brittle and low-strength materials, the diametric compression test may be listed [21]. It has been applied by Horabik et al. [22] for testing food powders.

The above text presents only a brief review of techniques intended for measuring the strength of bulk materials. The research in this field has been intensively developed over recent years due to increasing industry demand for easy-to-use and effective tools for measuring the mechanical properties of powders and granular solids. Although there are several methods to assess the flow properties of these materials, this topic is still unexhausted, and further research in this field is needed. Most of the existing methods have their own specifications and recommendations, as well as limitations in their application to certain materials or conditions. Therefore, there is a need to further develop the subject of caking and develop new measurement methods. In this study, a system for measuring the strength of loose food powders based on sliding up measuring rods made of various materials is presented. The advantage of the used device is its ability to measure the force during the consolidation of the material. An additional unique feature of the appliance is the ability to moisten the sample during the consolidation process. It is possible due to special, perforated cups that increase the material’s moisture absorption surface. The research has been carried out for potato starch and wheat flour, which constitute a large part of the global consumer market and play an important role in other branches of industry.

## 2. Materials and Methods

Potato starch (PPZ, Trzemeszno, Poland) and wheat flour type 500 (Młyn Szczepanki, Łasin, Poland) were used as materials in the experiment. Both powders were stored in airtight containers under room conditions and were not subjected to drying, moistening, or long storage times. The moisture contents were 9.5 ± 0.5% and 7.5 ± 0.5% for potato starch and wheat flour, respectively. Measurements of humidity were performed using a Mettler Toledo HG63 moisture analyzer (Laboratory and Weighing Technologies, Greifensee, Switzerland).

**Figure 1:**
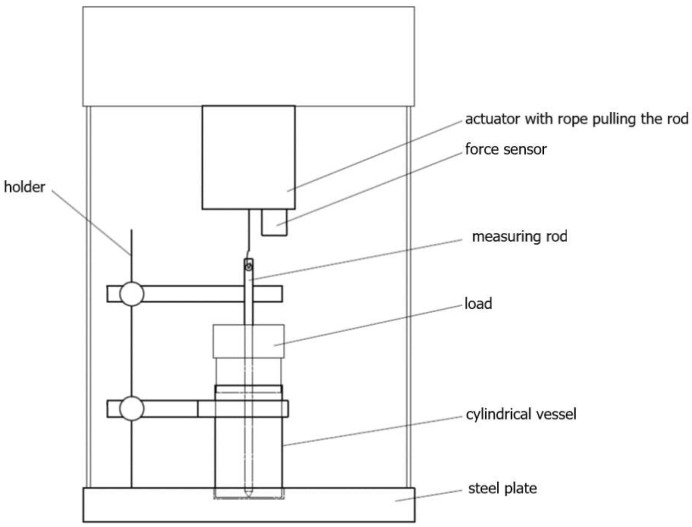
The pulled-based tester.

The pulled-based tester (Figure 1), consisting of a perforated, cylindrical vessel with a diameter of 24 mm, a height of 30 mm, and a mesh diameter of 1.5 mm, was used in the study. Inside the vessel, at the bottom, there is a thin cap, about 1.5 mm thick, with a centrally marked groove, enabling the measurement rods to be placed in the exact center of the sample. The measuring vessel is placed on a steel plate fixed to the device with a centering cutout. Next to the place intended for placing the measuring vessel, a holder is prepared to hold the measuring rod in a vertical position and to support the weight loading the sample. The measuring rods, made of stainless steel and polypropylene, have a height of 93.7 mm and a diameter of 4.82 mm. The rod diameter value was chosen based on similar experiments carried out with bulk materials [13,14,15], where a 5 mm diameter penetrometer was used. The prevalence of rod materials in equipment used in food powder processing dictated the selection of rod materials. Both types of rods have the form of a cylinder with a cone-shaped lower base that allows them to be centered in the centering washers placed in the measuring vessels and a hole cut in the upper part for connection to the force sensor. From the conical base, in each type of rod, there are conical incisions parallel to the base, which reach a height of approx. 33 mm of the length of the measuring rod. For each material of rod, two rods are made with different cutting depths (h), 1.3 mm and 0.65 mm.

**Figure 2:**
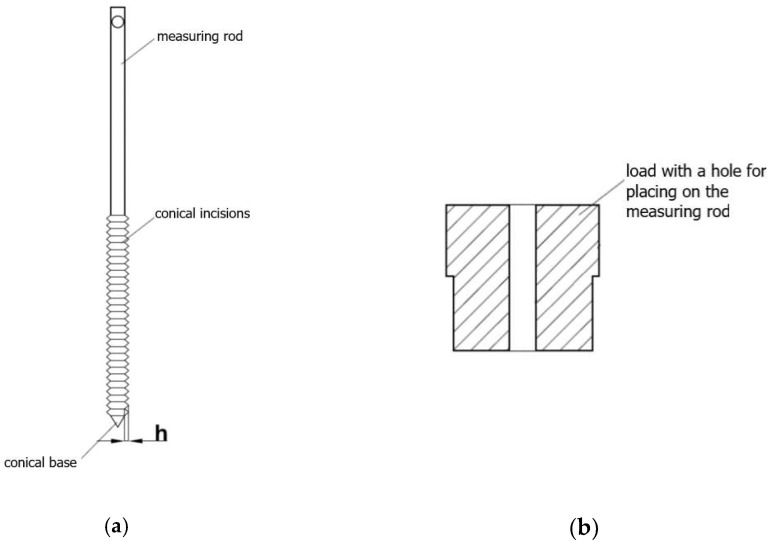
A scheme of the (**a**) measuring rod that is placed in a perforated container and (**b**) a load.

The perforated vessel is placed on the steel plate. The selected measuring rod, placed inside the vessel, is stabilized by the centering hole and the holder (Figure 2a). A vessel prepared in this way is filled with bulk material and then subjected to consolidation by vertical pressure induced by weight. Loads (Figure 2b) are placed on the measuring rod through the holes and move along it freely without friction. When the material is loaded, the measuring rod is centered and stably held by the test powder and, therefore, does not require further support by the holder. When the holder is removed, a rope is hooked at the top of the measuring rod. The second end of the rope is seated on an actuator-driven shaft. A load cell, also embedded in the stem, measures the value of the force transmitted by the rope in the range of 5 N. The actuator drives the pull cord with a pulling rate range of 1.75 to 9 mm/min. This paper presents results carried out using a speed of 1.75 mm/min, which gave the best repeatability and interpretability of the observed oscillations. With the help of the string and the set speed, the grooved part of the measuring rod is pulled out of the bulk material. As seen in the work of Fitzpatrick et al. [23], the highest force recorded by the sensor was taken as the strength of the agglomerate. The device is connected to a computer equipped with a program for carrying out and recording the performed tests. A computer program created in C++ and using the QtCreator 3.6.0 program allows for the recording of test results. The program allows users to detect the oscillations, count them, and calculate their frequency. It also allows users to record the force with which the test rod is pulled out of the sample.

Two load values were applied in the tests: 5 kPa and 10 kPa. The latter value, recommended by Eurocode 1 [24], is considered to be the maximum consolidation load for loose powders. This consolidation load has been applied by Stasiak et al. [25] to study the mechanical properties of potato starch.

**Figure 3:**
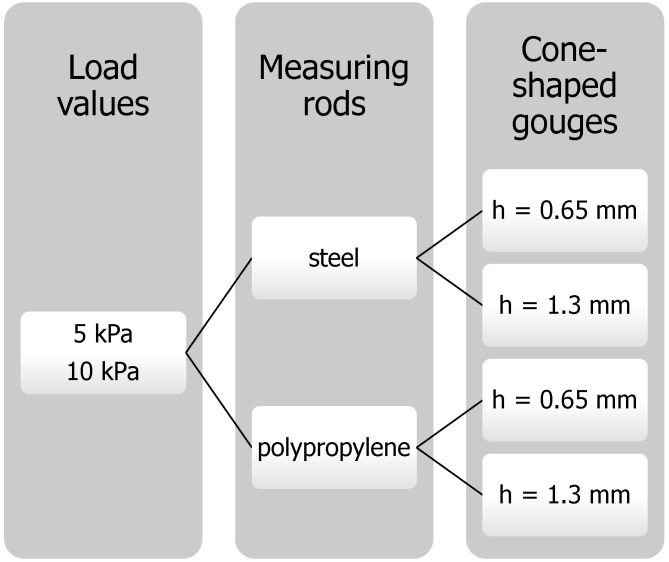
Variants of the experiment for each material.

Figure 3 shows variants of the experimental tests performed for each material. The samples were examined in 10 repetitions with the use of stainless steel and polypropylene measuring rods made with two groove depths, h = 0.65 mm and h = 1.3 mm. The evolution of the force with time was plotted, and the friction between the material and rod was calculated. The maximum force obtained in the first peak was determined from the resulting waveform plots. It was next divided by lateral unit area, providing the material strength, which is considered the caking strength of powders.

The average values of 10 repetitions were calculated together with standard deviations, and the analysis of variance was carried out using the software Statistica (v.13, StatSoft, Cracow, Poland).

## 3. Results and Discussion

**Figure 4:**
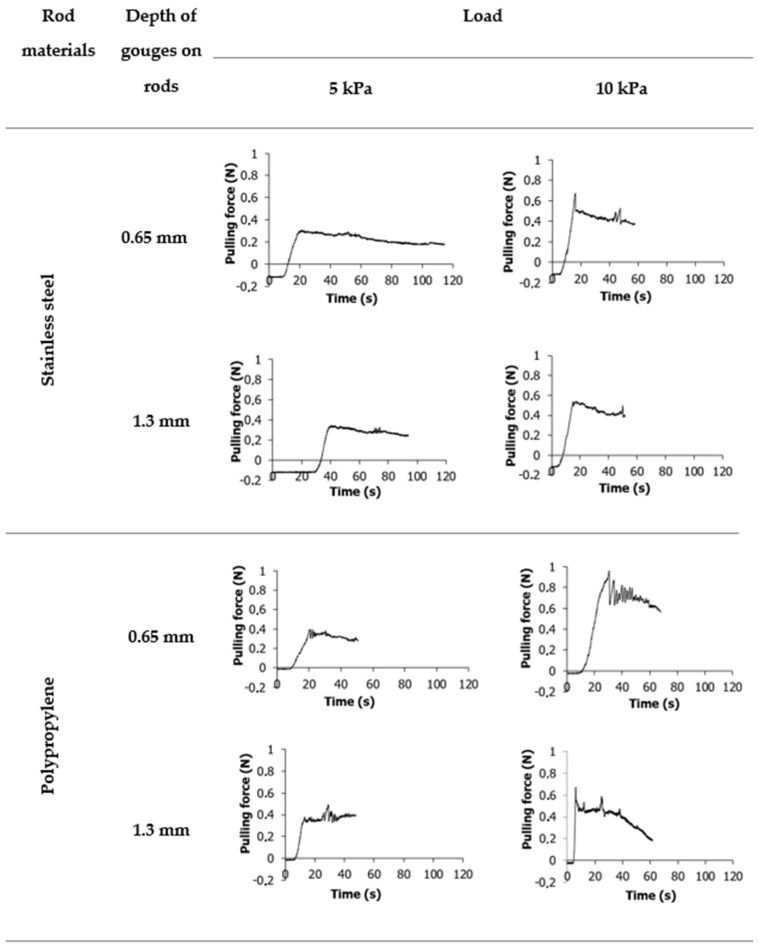
Example graphs plotted for wheat flour samples and two types of measuring rods. Graph values below zero at the start of the measurement are related to the taring of the measuring rod.

**Figure 5:**
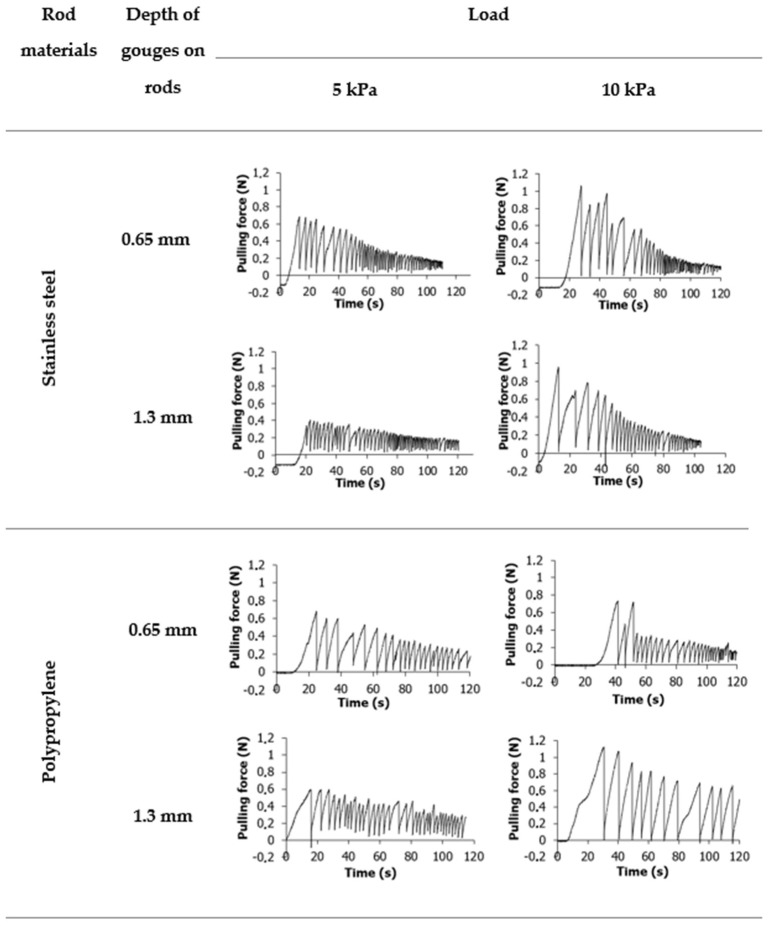
Example graphs plotted for potato starch samples and two types of measuring rods. Graph values below zero at the start of the measurement are related to the taring of the measuring rod.

The sensor recorded the force when the rods were pulled out. Figure 4 and Figure 5 show the evolution of the pulling force with time for wheat flour and potato starch, respectively, for two types of rods provided by the new tester. For both materials, the graphs start with a force value below zero. It is related to the calibration of the device and the subtraction of the mass of the test rods in order to get results strictly for the powder. In both cases, a sudden increase in strength followed by a decrease was observed. The maximum force is related to the strength of the material, while the decrease is conditioned by the decrease in strength caused by pulling out the rod. The curves obtained for wheat flour and potato starch are significantly different. In the case of wheat flour (Figure 4), a smooth increase in the force up to a certain value has been observed, followed by a slight decrease. The graphs for steel rod were smoother than for polypropylene rod. Figure 5 shows high oscillations of the pulling force in the potato starch. These observations corroborate results from shear tests performed for potato starch by other authors [6,26,27,28,29]. The oscillations of forces measured in some powders are associated with the slip-stick effect. This phenomenon occurs in many physical systems where surfaces are displaced relative to each other. The slip-stick friction leads to spontaneous, jerky movements or self-excited oscillations of constant or variable frequency [30]. The occurrence of oscillations is affected by the material’s compressibility. Oscillations may occur as a consequence of compaction and dilatation around the shear region in the material [31]. When the material is compacted, its strength increases. Hence, the material has a greater ability to withstand higher shear loads. When the maximum strength is exceeded, dilatation in the shear zone occurs, and strength decreases [26].

**Figure 6:**
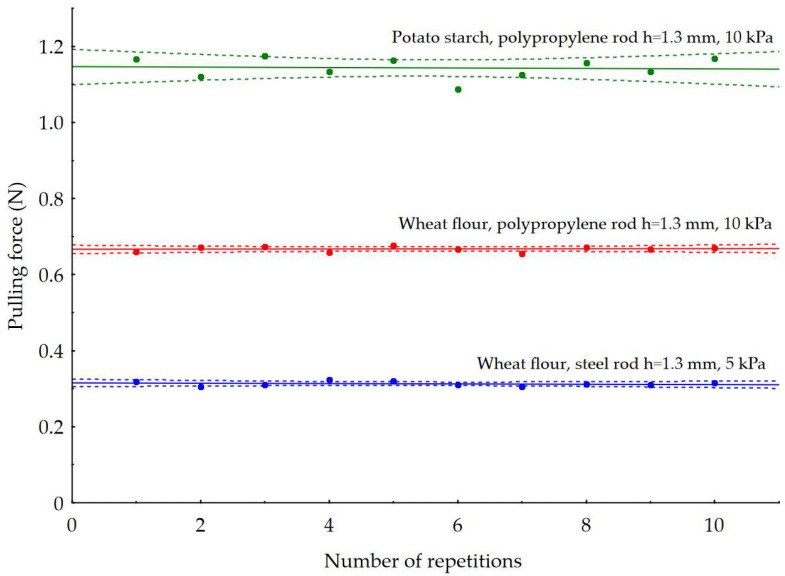
Maximum pull-out forces of rods from powder samples for ten repetitions. Circles indicate plotted values, a solid line indicates a match. The dotted lines represent the 0.95 confidence intervals.

Figure 6 presents a distribution of the maximum force values recorded in ten repetitions during the pulling out of rods from powder samples. The pulling forces on steel rods with a notch depth of h = 1.3 mm from a wheat flour powder sample at a load of 5 kPa were found to be in the range of 0.3–0.33 N. In this case, the coefficient of variation in the pulling force relative to the average is 5.93%. The pulling force ranges of the polypropylene rod with notches of h = 1.3 mm depth and a load of 10 kPa from the potato starch and wheat flour samples were 1.09–1.17 N and 0.66–0.68 N, respectively, for the instances shown in the graphs. In all the cases studied, the coefficient of variation in the pulling force did not exceed 10%. An average value of the pulling force has been calculated for all repetitions, which has been then converted to caking strength.

**Figure 7:**
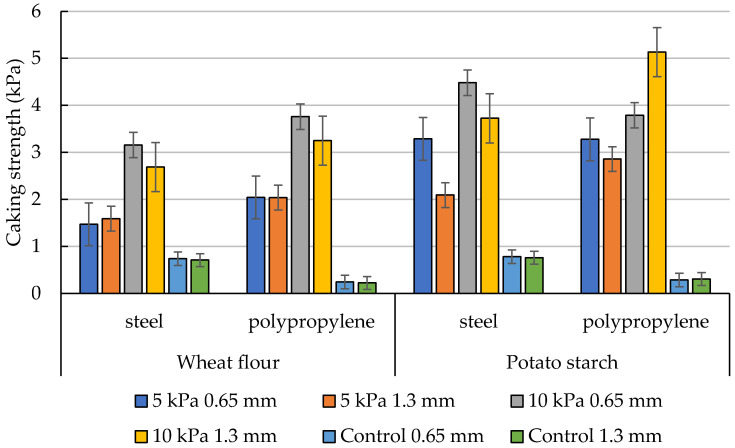
The cake strengths of wheat flour and potato starch for two types of measuring rods, along with error bars indicating the standard deviation.

Figure 7 presents the cake strengths for wheat flour and potato starch, measured for four types of measuring rods. The mean values are shown along with the error bars indicating the standard deviation. The lowest cake strengths have been obtained in control tests carried out without consolidation of the material. Control tests performed for polypropylene rods have provided strengths significantly lower than those obtained for steel rods. The opposite trend has been observed for consolidated samples, where the use of steel rods decreased the cake strength of the materials.

According to expectations, higher cake strengths have been obtained for samples consolidated with a maximal load of 10 kPa. It was due to the high compressibility of the material exposed to the highest force. In each measurement variant, lower values of strength have been obtained for wheat flour. It may result from the intrinsic properties of the material and its degree of compressibility. The strength of wheat flour is also determined by its morphological structure and particle size [27]. These results are in accordance with the results obtained by Wajs et al. [13,14] for the same materials subjected to the penetration test.

Regardless of the flour type, the values of cake strength measured with the polypropylene rod have been higher as compared to the steel rod. A greater sticking of particles of material to the surface of the steel rod has been observed. In the case of the polypropylene rod, the sticking of the powder particles has been smaller or has not occurred. This effect may be due to the adhesiveness of powders, which was found to be a function of relative humidity [32], as well as the influence of the surface roughness of the material rods. According to Lyashenko and Pohrt [33], the maximal magnitude of the attractive force (adhesion) is dependent on surface roughness. They have found that adhesion and detachment from rough surfaces happen more quickly based on observations of the development of the contact surface. Surface roughness is higher in stainless steel. It has a roughness coefficient of 0.015 mm, whereas plastic has a roughness coefficient of 0.0015 mm [34]. It is therefore conceivable that the steel rod’s material particles had a smaller adhesive surface, which contributed to the lower force readings when the needle was pulled from the powder samples. Both tested powders are characterized by high compressibility [27,31]. The packing of particles increases under load, resulting in a change in the steel-powder and plastic-powder interactions. The lower forces obtained for steel rods may be due to the fact that the adhesion force between the powder particles under load and steel is greater than the interparticle forces in the material. Thus, the results obtained may be the result of internal friction between the powder particles.

Food powders, such as wheat flour and potato starch, consistently change their strength properties with humidity, both in low and high loads [6]. Therefore, additional analyses excluding humidity are needed to find the reason for the above-mentioned effects.

In both powders, changes in strength have been observed to be dependent on the depth of the grooves on the rod. The application of needles with a groove depth of 0.65 mm has provided higher values of cake strength as compared to needles with notches 1.3 mm deep. The differences have been less pronounced for steel rods and wheat flour. This is due to the properties of the particles in that powder. Wheat flour particles have a lenticular shape, significant deformation on the surface, and are small in size, while potato starch particles are larger and more regular in shape. These properties may affect the orientation of the arranged particles, determining the mechanical properties of the material [27]. Hence, it is possible that during consolidation, the wheat flour particles packed more easily, and the depth of the grooves had no significant effect on the cake strength of this material. On the other hand, the more regular and larger starch particles did not fill the grooves to such a high degree.

**Figure 8:**
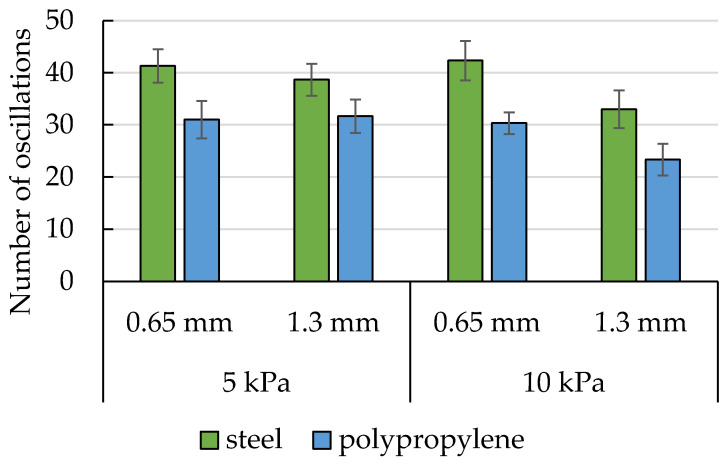
The average number of peaks recorded in potato starch graphs. The bars represent the standard deviation.

**Figure 9:**
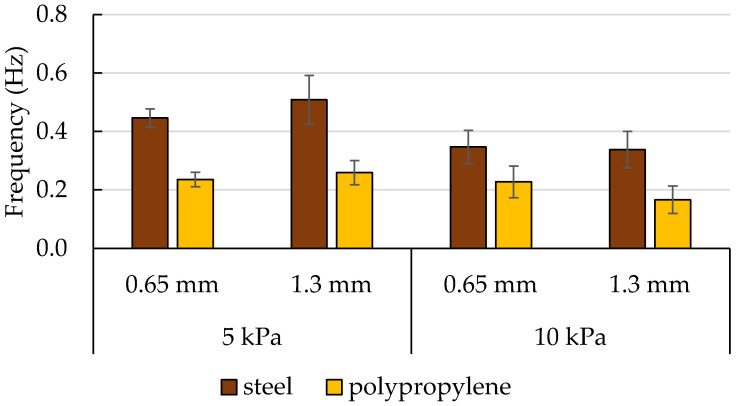
The mean frequency of oscillations of the pulling force in potato starch. The bars represent the standard deviation.

Additional analysis of the oscillations of the pulling force occurring in potato starch during measurements conducted with the pulled-based tester has been carried out. The average number of peaks (Figure 8) and their frequency (Figure 9) have been calculated within 120 s from the moment when the sensor force increased, or within a shorter time until the oscillation ceased. The rod pullout force from powders that had not been exposed to load consolidation has been used as the control test in this experiment. The force values in these samples had grown to insignificant levels before they decreased linearly. The experimental runs for the potato starch control samples have not revealed the existence of force oscillations. Therefore, the control samples are not included in Figure 8 and Figure 9.

The absence of oscillations in the untreated potato starch samples indicates the influence of vertical thrust on the slip-stick phenomenon. This means that on the surface of the stored material, the phenomenon will be minimal. As the pressure on the lower parts of the material increases, the risk of the slip-stick phenomenon increases. Figure 8 demonstrates that the material of the measuring rod had an effect on the occurrence of oscillations. The steel rod has produced a greater number of peaks, showing that this material facilitates the occurrence of the slip-stick phenomenon. It is probable that the roughness of the rod affected the existence of oscillations in this situation as well. In rough materials, particle detachment occurs more rapidly [33].

Figure 9 indicates the dependence of the frequency of oscillations of force on the material of the rod. The frequency of oscillations of force measured with the polypropylene rod has been smaller. Neither the depth of the indentations nor the load consolidation have been found to affect the frequency values. The oscillations related to the slip-stick effect cause difficulties in the interpretation of results. The oscillations in force in starches and dextrins at loads of 4, 6, and 10 kPa have also been reported by Stasiak et al. [9]. These authors have observed that the higher the load, the earlier the slip-stick phenomenon appeared, with oscillation amplitudes reaching the order of about 3 kPa. In the work by Molenda et al. [27], the oscillations resulting from the slip-stick friction have been observed in wheat flour; however, their frequency was lower than that in potato starch, and the oscillations were less regular. One of the factors affecting the frequency of oscillations with a pattern described as ‘saw tooth’, which also concerns the presented results, is a shear rate. In the common shear velocity range of approximately 1 to 2 mm/s, frequencies in the range of 1 to 10 Hz are often observed [30].

## 4. Conclusions

A pulled-based tester for the determination of the food powder’s strength has been used in this study. A series of tests have been conducted for wheat flour and potato starch with high repeatability, proving the applicability of the new device for measuring the degree of caking of food powders. Along with the good repeatability, the pulled-based tester is characterized by fast measurement and easy analysis of the results, which is an extremely important aspect when selecting the right parameters for processing. A series of tests on wheat flour and potato starch with high reproducibility have confirmed the applicability of the new device for measuring the degree of agglomeration of food powders. This research has resulted in two patents being granted at the Patent Office of the Republic of Poland (P.436420 and P.436421).

The device allows for the measurement of the rod’s pull-out force when the material is loaded. The device’s capability to moisten the sample during the consolidation process is another distinctive feature. This is achievable because the vessel’s perforated walls increase the material’s surface area for water absorption. Due to the holes in the measurement vessel’s walls, the powder comes into increased contact with the environment. This is an original solution compared to the caking and strength testing methods available in the literature.

The tests performed for potato starch and wheat flour have provided significantly different temporal evolutions of the pulling force. These differences may be due to the intrinsic properties of the examined powders. The analysis of the pulling force oscillations in potato starch allowed slip-stick friction to be identified in this material. Thus, the new device can also be used to characterize the slip-stick phenomenon in powders of different botanical origins.

Regardless of the type of powder, the strength values measured with the polypropylene rod were higher compared to the steel. This effect may be due to the adhesion of the powder and the influence of the surface roughness of the rod material. The differences in cake strengths of tested materials for two groove depths of the measuring rods may be due to differences in the morphological structure of powder particles. However, further analyses for materials with different morphological structures should be carried out to verify the contribution of the depth of the grooves to the cake strength of the material. In the future, additional time-consolidation measurements will be carried out using the new device to get a more detailed insight into the effect of moisture and load consolidation on the caking ability of food powders. In the future study, different relative humidity conditions will be considered, and the consolidation of samples in perforated dishes will be conducted to assess the influence of humidity on the caking process.

## Data Availability

Data sharing is not applicable.
